# Intravoxel Incoherent Motion Diffusion Weighted MR Imaging at 3.0 T: Assessment of Steatohepatitis and Fibrosis Compared with Liver Biopsy in Type 2 Diabetic Patients

**DOI:** 10.1371/journal.pone.0125653

**Published:** 2015-05-11

**Authors:** Daniella Braz Parente, Fernando Fernandes Paiva, Jaime Araújo Oliveira Neto, Lilian Machado-Silva, Fatima Aparecida Ferreira Figueiredo, Valeria Lanzoni, Carlos Frederico Ferreira Campos, Pedro Emmanuel Alvarenga Americano do Brasil, Marilia de Brito Gomes, Renata de Mello Perez, Rosana Souza Rodrigues

**Affiliations:** 1 D’Or Institute for Research and Education, Rio de Janeiro, Brazil; 2 Federal University of Rio de Janeiro, Rio de Janeiro, Brazil; 3 University of the State of Rio de Janeiro, Rio de Janeiro, Brazil; 4 Institute of Physics of São Carlos, University of São Paulo, São Carlos, Brazil; 5 Federal University of São Paulo, São Paulo, Brazil; University of Modena & Reggio Emilia, ITALY

## Abstract

**Objective:**

To evaluate the capability of intravoxel incoherent motion (IVIM) diffusion-weighted imaging (DWI) to assess steatohepatitis and fibrosis determined by histopathology in type 2 diabetic patients.

**Methods:**

Fifty-nine type 2 diabetic patients (49 women, 10 men; mean age, 54 ± 9 years) were submitted to liver biopsy for the evaluation of non-alcoholic fatty liver disease (NAFLD) and underwent DWI on a 3.0T MR system using 10 b values. Institutional approval and patient consent were obtained. Pure molecular-based (D), perfusion-related (D*), and vascular fraction (f) were calculated using a double exponential model and least squares curve fitting. D, D*, and f were compared between patients with and without steatohepatitis and between patients with and without fibrosis. The variables were compared by using the Ranksum test and Student t-test.

**Results:**

Steatohepatitis was observed in 22 patients and fibrosis in 16 patients. A lower D median (0.70 s/mm^2^ vs. 0.83 s/mm^2^, p<0.05) and a lower D* median (34.39 s/mm^2^ vs. 45.23 s/mm^2^, p<0.05) were observed among those with steatohepatitis. A lower D median (0.70 s/mm^2^ vs. 0.82 s/mm^2^, p<0.05) and a lower D* median (35.01 s/mm^2^ vs. 44.76 s/mm^2^, p=0.05) were also observed among those with fibrosis.

**Conclusion:**

IVIM-DWI has the potential to aid in the characterization of steatohepatitis and fibrosis.

## Introduction

Non-alcoholic fatty liver disease (NAFLD) is a clinicopathologic syndrome that varies from isolated steatosis, to steatohepatitis, which may progress to fibrosis and cirrhosis, with the risk for development of hepatocellular carcinoma [1–3]. NAFLD is a major public health problem with increasing incidence that affects up to one third of the population in all age groups and ethnicities. According to epidemiology data, NAFLD will continue to be a leading cause of chronic liver disease in the next decades [2,4,5]. Its main risk factors are obesity and type 2 diabetes, also with increasing prevalences [2,4,5].

The prevalence of NAFLD in type 2 diabetic patients is around 70% [6–8], with a more aggressive course in this group [9–12]. The prevalence of non-alcoholic steatohepatitis (NASH) in diabetics is not well established and has been estimated to be between 22% and 88% [7,13,14]. Cirrhosis develops in 15% to 25% of patients with NASH and 30% to 40% of cirrhotic patients have their death related to chronic liver disease in 10 years [1,2,15]. Currently, it is only possible to differentiate between the various forms of presentation of NAFLD (isolated steatosis, steatohepatitis and/or fibrosis) by liver biopsy, an invasive method that cannot be used to screening or to follow-up [1,3,16,17]. Due to limitations of liver biopsy (sampling and observer variations) and the need for staging of NAFLD, a lot of research have been done to develop non-invasive methods for the detection of inflammation and fibrosis, based on a combination of serum markers such as NASH test and Cytokeratin-18 for the diagnosis of steatohepatitis, and NAFLD-fibrosis score, BARD score, FIB-4, and HEPA score for the diagnosis of fibrosis. Unfortunately, none of them are accurate enough and they are not validated for clinical use [18,19]. Fibroscan is a method that measures liver stiffness and is good for the identification on non-significant fibrosis (F0 and F1) and advanced fibrosis (F4), although overlapping results are seen in F2 and F3 groups [19,20]. Thus, the identification of a non-invasive method that allows early detection of inflammation and fibrosis that are markers of more severe disease, to stage the disease, and to follow these patients is extremely important.

Previous studies indicate diffusion weighted imaging (DWI) as a tool for the evaluation of inflammation and fibrosis, where a lower ADC (apparent diffusion coefficient) value indicates a tendency to increased inflammation and/or fibrosis [21–28]. Recently, some studies have used intravoxel incoherent motion (IVIM) diffusion-weighted magnetic resonance (MR) imaging but these studies compared only advanced fibrosis (cirrhosis—F4 with controls. However, most of these studies were performed in 1.5T magnets, using a low number of patients, with varied number and values of b-values and have controversial results. There are scarce data regarding IVIM DW performance in steatohepatitis. One experimental study evaluated the influence of steatohepatitis on IVIM diffusion and showed reduction on perfusion fraction (f component) in rabbits with steatohepatitis compared to controls, with excellent area under the ROC curve values [29]. The sole study on humans that evaluates steatohepatitis and IVIM diffusion imaging using histopathology as reference was performed without respiratory triggering and used only 3 b values (0, 100, and 500 s/mm^2^). The absence of multiple low b values precluded the calculation of the perfusion-related diffusion (D*) [30].

The purpose of this study was to evaluate the capability of the respiratory-triggered diffusion-weighted MR imaging analysis to assess steatohepatitis and fibrosis determined by liver biopsy in type 2 diabetic patients when IVIM theory is taken into account using 10 b values.

## Materials and Methods

### Patients

This prospective study was approved by our institutional review board and written informed consent was obtained from all patients. Type 2 diabetic subjects from the endocrinology department of the University of the State of Rio de Janeiro, Brazil, between 18 and 70 years of age with a clinical indication for liver biopsy for the evaluation of NAFLD were consecutively enrolled between June 2010 and February 2012. Patients with other possible causes for chronic liver disease (i.e., viral hepatitis, alcoholism), severe cardiopulmonary disease, renal failure, coagulopathies, use of medications that could cause NAFLD, and patients with contraindications to MRI (i.e., claustrophobia, metallic implants) or patients that did not underwent liver biopsy were excluded. Patients with other etiology for chronic liver disease at liver biopsy, insufficient biopsy fragment for histological analysis, or low-quality IVIM diffusion weighted MRI were withdrawal.

### Diffusion-weighted imaging

All patients underwent respiratory-triggered, fat-suppressed, echo-planar diffusion-weighted MR imaging acquired on a 3.0T Philips Achieva MR system (Philips Medical Systems, Eindhoven, Netherlands) with a QUASAR Dual gradient system with peak gradient amplitude of 80 mT/m and slew rate of 200 mT/m/ms. Ten b-values were used (0, 10, 20, 40, 80, 160, 200, 400, 800, and 1000 s/mm^2^). A whole body coil was used as a transmitter, while a sixteen-element receiver-only phased-array coil was used for signal reception. Field of view was 400 x 300 mm; slice thickness, 6mm; interslice gap, 8 mm, repetition time, 1 respiratory cycle; echo time, 72 ms; and number of slices, 6.

All patients were supine. Patients were instructed to breathe smoothly for the respiratory-triggered sequences. The DWI acquisition time was approximately 3 minutes, dependent on patient breathing regularity. T2 weighted images on the coronal and axial planes were performed for anatomical reference.

### Image Interpretation—Data Analyses

**Fig 1:**
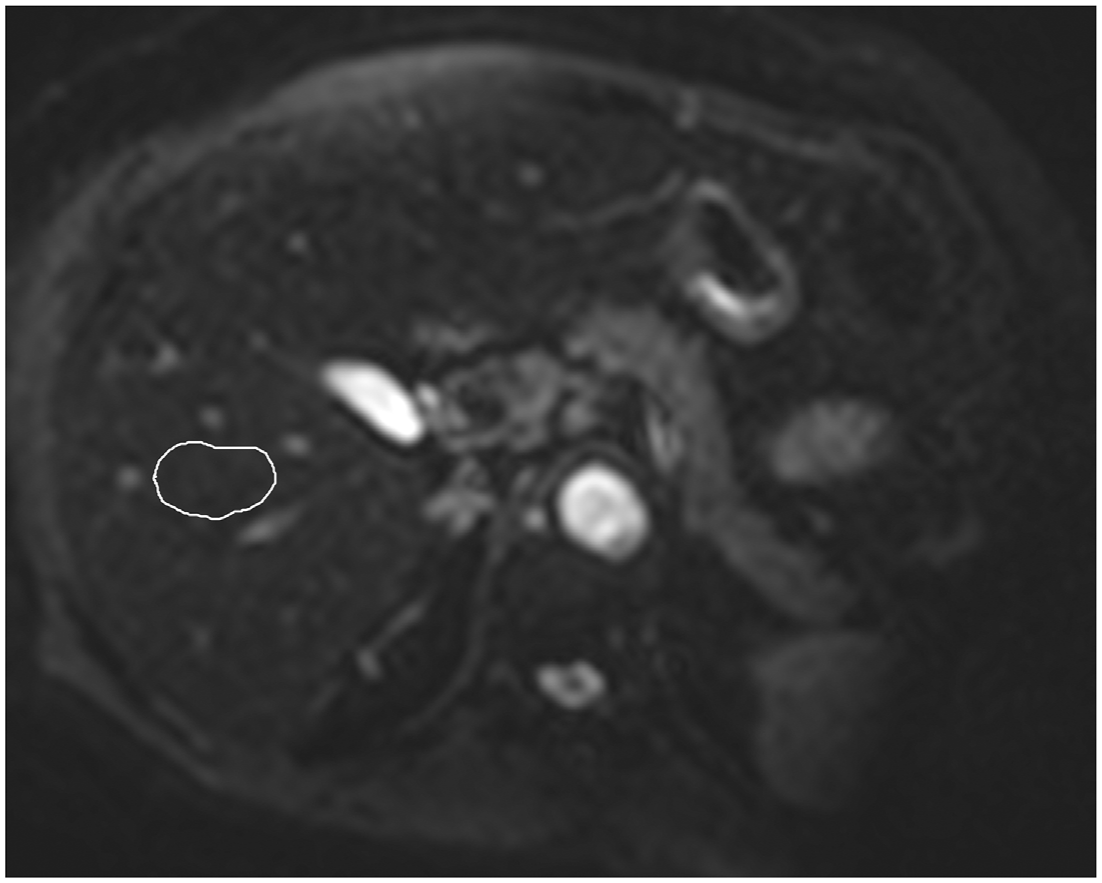
IVIM diffusion-weighted MR image (b = 0 s/mm^2^) from a 62 year-old woman with type 2 diabetes and steatohepatitis. ROI was manually drawn at segment V, as shown (in the same region where biopsies were performed). This is a representative figure to demonstrate ROI positioning at the liver. Diffusion images have low signal-to-noise ratio, therefore the “blurred” appearance. IVIM, intravoxel incoherent-motion; MR, magnetic resonance; ROI, region of interest.

All MR imaging was transferred to the workstation (View Forum; Philips). One radiologist (D.B.P., 10 years of experience with liver imaging) manually drew a region of interest (ROI) approximately 900 mm^2^ in area at segment V of the liver. The signal intensity (SI) in the region of interest (ROI) was recorded for each b-value. The copy-and-paste function was used to propagate the ROI to all images (Fig 1). This location corresponded to the region of the liver biopsies.

Pure molecular-based (D), perfusion-related (D*) and vascular fraction (f) were calculated using a double exponential model described by the following equation [31].

$$\frac{\text{S}}{\text{S}0}=\left(1-\text{f}\right){\text{ e}}^{-\text{bD}}+{\text{f e}}^{-{\text{bD}}^{\text{*}}}$$ (1)

To improve robustness in the fitting process, a two-steps analysis was employed as it has been done in previously published reports [26,32]. Since D* is typically 10 times greater than D [33], its influence on the signal decay for b-values greater than 200 s/mm^2^ is negligible. In that case, Eq 1 can be simplified and D can be estimated by a linear fit:

$$\frac{S}{S^{\prime }}=e^{-bD}$$ (2)

where S’ allows the vascular fraction to be determined according to the following equation:

$$f=\frac{\left(S_{0}-S^{\prime }\right)}{S_{0}}$$ (3)

Once both D and f have been determined by using Eqs 2 and 3, a constrained least squares curve fitting was done based on Eq 1 to calculate D*.

### Liver Biopsy and Evaluation

All patients underwent subcostal liver biopsy of the right lobe with ultrasound (US) guidance by using a 16-gauge Menghini biopsy needle. Specimens measuring 2 cm in length or longer were fixed in 10% formaldehyde solution and embedded in paraffin. The sections were then stained with hematoxylin eosin, Masson’s trichrome, and Perls stains. Patients with insufficient specimens or with other cause for chronic liver disease were withdrawal.

One pathologist (V.L., 28 years of experience) prospectively examined all biopsy slides. The extent of steatosis was evaluated semi-quantitatively assessing the percentage involvement by steatotic hepatocytes in liver parenchyma: 0–33%—mild; 33–66%—moderate, and > 66%- severe [1]. Steatohepatitis was present when variable degrees of steatosis were accompanied by mixed-cell inflammatory infiltrates in the hepatic lobules and damage of hepatocytes [34]. The late was characterized by contiguous patch of hepatocyte showing prominent ballooning, typically in zone 3, with or without apoptosis and necrosis, and with or without fibrosis, scored from F0 to F4 (F0, absent; F1, perisinusoidal or portal/periportal fibrosis; F2 zone 3 perisinusoidal fibrosis and periportal fibrosis; F3, bridging fibrosis; F4, cirrhosis) [35]. Pathologist also independently graded liver biopsies according to the NASH Clinical Research Network Scoring System [35].

### Statistical Analysis

Statistical analyses were conducted using R-project for statistical computing software (version 3.1.2). Pure molecular diffusion (D), perfusion-related diffusion (D*), and perfusion fraction (f) parameters were compared between patients with and without steatohepatitis, with and without fibrosis, different degrees of fibrosis and different degrees of steatosis. The variables were compared by using either Student t-test, ANOVA or Ranksum test. The accuracy was estimated by empirical receiver operating characteristics’ (ROC) area under the curve (AUC) using the trapezoidal method. Decision thresholds were estimated for each parameter through the maximization of the Youden J index of several points on a robust smoothed curve. P values ≤ 0.05 were considered to be significant.

## Results

### Patients

A total of 80 patients were enrolled in this study. No patient had contraindications to MR imaging. Five patients were excluded: four due to refusal of the liver biopsy and one for precordial pain that precluded biopsy. Sixteen patients were withdrawal: one patient due to an insufficient amount of biopsied tissue, one for granulomatous hepatitis upon histological analysis, and fourteen for low-quality IVIM diffusion weighted MRI. Thus, the study evaluated 59 patients, including 49 (83%) women and 10 (17%) men with a mean age of 54 ± 9 years. The mean body mass index was 31.5 kg/m^2^ (range, 23.8–42.7 kg/m^2^). Considering a mean body mass index (BMI) between 20 and 25 kg/m^2^ to be normal, 97% (57 of 59) of the patients were overweight (BMI > 25 kg/m^2^) and 63% (37 of 59) were obese (BMI > 30 kg/m^2^). Diffusion-weighted imaging was performed within 3 months from the liver biopsy. Thirty-three percent of all patients had elevated aminotransferase levels.

### Histological Analysis

At histopathologic examination, the incidence of hepatic steatosis was 92%. Thirty-one patients (53%) had mild steatosis, 6 (10%) had moderate steatosis, and 17 (29%) had severe steatosis. Thirty-seven percent of patients (22 of 59) had steatohepatitis. Fibrosis was observed in 27% of patients (16 of 59). The breakdown of NAFLD fibrosis scores among these patients were: F1, 9 patients; F2, 5 patients; F3, 1 patients; and F4, 1 patient.

### Diagnostic Accuracy for the Diagnosis of NASH and Fibrosis

**Fig 2:**
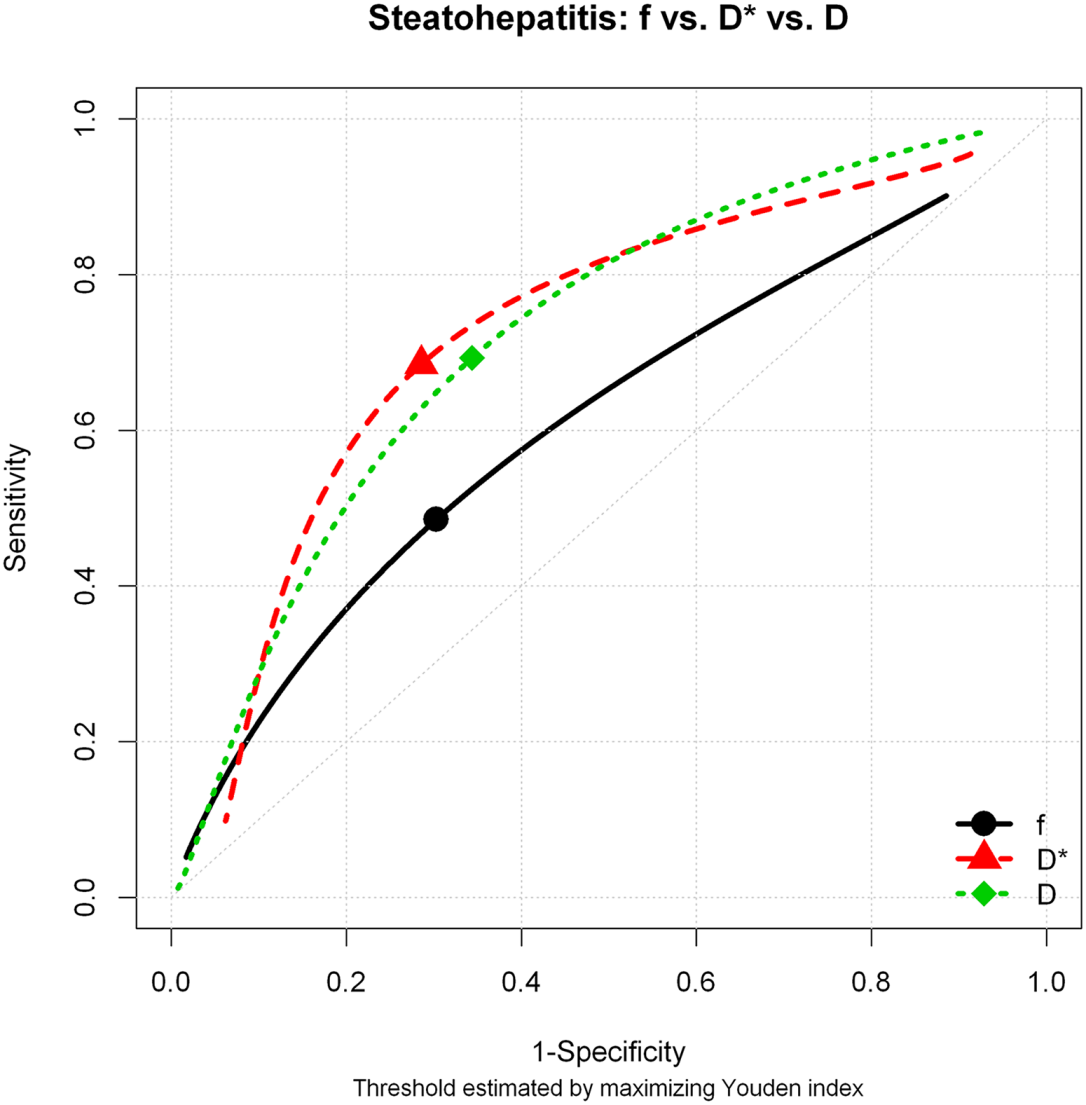
Diagnostic performance for D, D*, and f for the diagnosis of steatohepatitis using histopathology as the gold standard. The best cut-off point was identified using the Youden index.

The comparison between patients with and without steatohepatitis showed statistically significant difference for the pure molecular diffusion (D) and for perfusion-related diffusion (D*), with p values 0.002 and 0.023, respectively. No significant difference was observed for the perfusion fraction (f). These results are summarized in Table 1. The areas under the curve for D, D* and f were 0.742, 0.678 and 0.607, respectively (Fig 2). The diagnostic performance for D, D*, and f is shown in Table 2.

**Table 1:** Comparison of D, D*, and f between patients without vs. with NASH and between patients without vs. with fibrosis (F0 vs. F1–F4).

NASH				
	Total	No NASH	NASH	p value
**Number of patients**	59	37	22	
**f**				
**Mean**	36.46	37.89	34.05	0.131
**(SD)**	(9.42)	(8.6)	(10.42)	
D* (x10 ^**-3**^ **mm** ^**2**^ **/s)**				
**Median**	40.88	45.23	34.39	0.024
**(IQR)**	(32.64,51.47)	(38.01,56.05)	(24.5,45.51)	
**D (x10** ^**-3**^ **mm** ^**2**^ **/s)**				
**Median**	0.76	0.83	0.70	0.002
**(IQR)**	(0.68,0.86)	(0.74,0.88)	(0.62,0.76)	
**Fibrosis**				
	**Total**	**No fibrosis**	**Fibrosis**	**p value**
**Number of patients**	59	43	16	
**f**				
**Mean**	36.46	37.71	33.08	0.094
**(SD)**	(9.42)	(8.2)	(11.75)	
D* (x10 ^**-3**^ **mm** ^**2**^ **/s)**				
**Median**	40.88	44.76	35.01	0.051
**(IQR)**	(32.64,51.47)	(35.25,54.26)	(23.97,43.49)	
**D (x10** ^**-3**^ **mm** ^**2**^ **/s)**				
**Median**	0.76	0.82	0.70	0.025
**(IQR)**	(0.68,0.86)	(0.70,0.88)	(0.56,0.78)	

D, pure molecular diffusion; D*, perfusion-related diffusion; f, vascular fraction; NASH; non-alcoholic steatohepatitis; SD; Standard deviation; IQR; interquartil range

**Table 2:** Comparison among D, D*, and f for the detection of NASH and fibrosis.

NASH				
	Cut-Off	Sensitivity	Specificity	AUC
**D**	0.760	0.693	0.656	0.742
D*	41.45	0.685	0.714	0.678
**f**	34.23	0.485	0.697	0.607
**Fibrosis**				
	**Cut-off**	**Sensitivity**	**Specificity**	**AUC**
**D**	0.730	0.589	0.680	0.692
D*	37.75	0.618	0.709	0.667
**f**	34.83	0.575	0.727	0.618

NASH, non-alcoholic steatohepatitis; D, pure molecular diffusion; D*, perfusion-related diffusion; f, perfusion fraction; AUC, area under the curve

**Fig 3:**
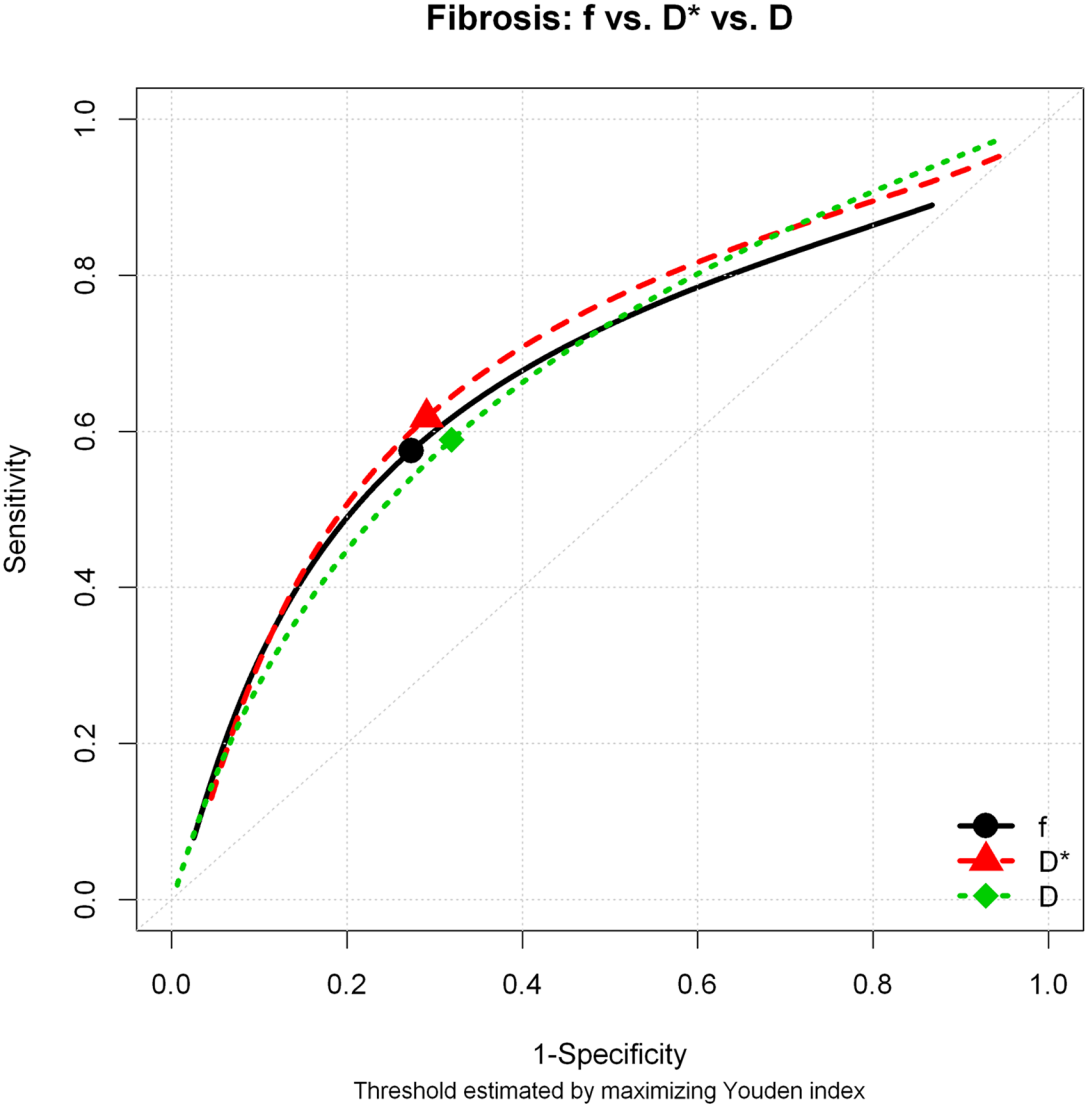
Diagnostic performance for D, D*, and f for the diagnosis of fibrosis using histopathology as the gold standard. The best cut-off point was identified using the Youden index.

The comparison between patients with and without fibrosis (F1–F4 vs. F0) showed statistically significant difference for the pure molecular diffusion (D) and for perfusion-related diffusion (D*), with p values 0.025 and 0.05, respectively. No significant difference was observed for the perfusion fraction (f). These results are summarized in Table 1. The areas under the curve for D, D* and f were 0.692, 0.667 and 0.618 and the cut-offs 0.730, 37.75, and 34.83, respectively. The diagnostic performance for D, D*, and f is shown in Fig 3 and Table 2.

Pure molecular diffusion (D), for perfusion-related diffusion (D*), and perfusion fraction (f) were distributed according to histological fibrosis stage groups (F0 to F4), as shown in Table 3. Although there is progressive decrease of D, D*, and f according to fibrosis severity, the sample of the group F3 and F4 was too small (2 patients) to allow a statistical comparison among the groups.

**Table 3:** Pure molecular diffusion (D), perfusion-related diffusion (D*), and perfusion fraction (f) in histological fibrosis stage groups (F0 to F4).

	F0	F1	F2	F3 or F4	Statistical test	p Value
**Number of patients**	43	9	5	2		
**f**						
**Mean**	37.03	37.41	34.83	23.09	Sample too small: group F3 or F4	NA
**(SD)**	(8.18)	(12.32)	(9.76)	(2.08)		
D* (x10 ^**-3**^ **mm** ^**2**^ **/s)**						
**Median**	40.88	42.48	34.2	32.08	Sample too small: group F3 or F4	NA
**(IQR)**	(33.75,54.26)	(25.99,51.11)	(32.64,35.83)	(24.85,39.3)		
**D (x10** ^**-3**^ **mm** ^**2**^ **/s)**						
**Median**	0.82	0.76	0.7	0.8	Sample too small: group F3 or F4	NA
**(IQR)**	(0.68,0.88)	(0.54,0.84)	(0.65,0.7)	(0.77,0.82)		

D, pure molecular diffusion; D*, perfusion-related diffusion; f, vascular fraction; SD; Standard deviation; IQR; interquartil range; NA, not assigned

Pure molecular diffusion (D), for perfusion-related diffusion (D*), and perfusion fraction (f) were also distributed according to different degrees of steatosis (Table 4). A significant decrease of pure molecular diffusion (D) was shown as steatosis increases (p < 0.005). Although a significant decrease could not be demonstrated for perfusion-related diffusion (D*) as steatosis increases, the results point to a tendency (p = 0.10). No significant difference was noted for perfusion fraction (f).

**Table 4:** Pure molecular diffusion (D), perfusion-related diffusion (D*), and perfusion fraction (f) in different degrees of steatosis.

	No Steatosis	Mild Steatosis	Moderate Steatosis	Severe Steatosis	Statistical test	p value
**Number of patients**	5	31	6	17		
**f**						
**Mean**	39.59	36.2	34.18	36.78	ANOVA F-test (3, 55 df) = 0.305	0.8217
**(SD)**	(14.04)	(8.07)	(7.06)	(11.21)		
D* (x10 ^**-3**^ **mm** ^**2**^ **/s)**						
**Median**	48.07	45.88	39.91	35.01	Kruskal-Wallis test	0.1056
**(IQR)**	(40.88,48.22)	(35.64,63.79)	(26.21,47.5)	(27.21,42.2)		
**D (x10** ^**-3**^ **mm** ^**2**^ **/s)**						
**Median**	0.96	0.83	0.75	0.7	Kruskal-Wallis test	0.002
**(IQR)**	(0.92,0.97)	(0.74,0.88)	(0.68,0.81)	(0.58,0.76)		

D, pure molecular diffusion; D*, perfusion-related diffusion; f, vascular fraction; SD; Standard deviation; IQR; interquartil range.

## Discussion

Steatohepatitis and/or fibrosis evaluated by histology are prognostic markers of severity in NAFLD patients. This study evaluates the role of respiratory triggered IVIM diffusion weighted imaging with multiple b values as a non-invasive diagnostic method for detection of steatohepatitis and/or fibrosis in humans using liver biopsy as the reference standard. Our results suggest that D and D* could be useful to identify NASH and/or fibrosis in diabetic patients.

Pure molecular diffusion (D) can be reduced in NASH, affected by the structural changes that occur in the liver. In steatohepatitis, there is accumulation of fat in hepatic cells, inflammation, and hepatocellular ballooning. Lobular inflammation and deposition of collagen fibers can also be present and reduce the Brownian motion of water molecules. The perfusion component (D*) and the vascular volume fraction (f) reflect the changes that occur in the liver blood flow and can also be decreased in NASH. The distortion of the microcirculatory anatomy and the compression of the sinusoidal space that occur in steatohepatitis can lead to changes in the liver blood flow [1].

The influence of steatohepatitis in the components of diffusion are not well known yet. Our results demonstrated that pure molecular diffusion (D) and the perfusion component of diffusion (D*) were significantly lower in patients with steatohepatitis. The perfusion fraction (f) was also lower in patients with steatohepatitis, but did not reach statistical significance. D and D* sensitivities, specificities, and AUC for the differentiation between the groups with and without steatohepatitis were 0.693 and 0.685, 0.656 and 0.714, and 0.742 and 0.678, respectively. A recent experimental study with rabbits achieved high diagnostic performance using the vascular fraction (f) to differentiate between the groups with progressive NAFLD severity with AUC higher than 0.900 [29]. Although Joo et al. [29] showed a high accuracy for the diagnosis of NASH, it has been already shown that the obtained diffusion parameters depend on the technical parameters which may hamper the comparison between results conducted in different experimental settings. The only human study on IVIM diffusion (b values 0, 100, 500 s/mm^2^) for NAFLD that used histology as the reference standard was not able to show any significant difference on D or f for NASH. This study did not evaluate D* [30]. Thus, it is important to reinforce that in order to make this a tool for noninvasive assessment of NAFLD and NASH, more validation studies should be performed in animals and humans [36] and further efforts need to be made to standardize the acquisition and postprocessing methods of the IVIM diffusion—weighted MR images. Further than that, since signal-to-noise ratio is a known issue for IVIM accuracy, Joo et al. [29] results may indicate that the technique can benefit from higher magnet field.

Progressive fibrosis and ultimately cirrhosis may occur in the liver during the course of NAFLD. In the fibrotic process, there is deposition of collagen molecules, glycosaminoglycans, and proteoglycans in the extracellular space of the liver that leads to restrictive barriers and may decrease pure molecular diffusion (D) of water. Throughout this process, liver circulation is also impaired. The deposition of collagen and other macromolecules in perisinusoidal space and the closure of fenestrations along the endothelium determine compression of sinusoids and resistance to sinusoidal blood flow with consequent microcirculatory perfusion reduction. Additionally, a progressive increase in the arterial vascularization and reduction in the portal blood flow also influences the perfusion component (D*) and the vascular fraction (f) [22,37].

Although mild fibrosis (F1 and F2) was observed in 88% of the population that had fibrosis, our results showed that pure molecular diffusion (D) and the perfusion component of diffusion (D*) were significantly lower in patients with fibrosis. The perfusion fraction (f) did not reach statistical significance but may point to a tendency, as f is lower in the group with fibrosis, with a p value of 0.09. D and D* sensitivities, specificities, and AUC for the differentiation between the groups with and without fibrosis were 0.589 and 0.618, 0.680 and 0.709, and 0.692 and 0.667, respectively.

In this study, although a progressive decrease of D, D*, and f was observed as the fibrosis stage increases, the sample of the group F3 and F4 was too small for a statistical comparison. Thus, fibrosis stage may have some interference on the values of D, D*, and f. However, from the practical point of view, fibrosis' degree of interference is secondary from a detection/prediction point of view. In clinical practice, the phenomena will occur concurrently, as patients will often have simultaneously different degrees of steatosis, steatohepatitis, and fibrosis. Moreover, the degree of fibrosis as determined by histopathology will not be available prior to MR imaging. Even if the fibrosis effect is large on NASH prediction by D, D*, and f, the overall accuracy interpretation remains the same. Although D and D* were significantly reduced in patients with fibrosis, more studies are necessary to validate IVIM DWI before it is incorporated in the clinical practice.

The few studies that evaluated IVIM diffusion for the diagnosis of fibrosis enrolled populations with advanced disease and have controversial results [25,26,38,39]. The two experimental studies with different stages of fibrosis also had controversial results [40,41]. Most find reduction in one of the components of IVIM diffusion, but the affected component varies among the different studies. While some find reduction on D [26,38,40], others find reduction on D* [25,26,39,40], and others on f [26,38,41]. A possible explanation for these controversial results is that all components of IVIM diffusion may be affected by fibrosis. Pure molecular diffusion (D) is reduced because of the architectural changes, and the perfusion component (D*) and the vascular fraction (f) are lower because of blood flow disturbances that occur in fibrosis. Further than that, as already mentioned, a standardization of the acquisition and post-processing methods of the IVIM data is also required in order to allow further comparison between the results from different studies and may be addressed in future studies.

There is still debate on the influence of steatosis as a confounding factor in IVIM diffusion components. While some authors have shown that steatosis reduces D, D*, and f compared to normal liver [42,43], others show that steatosis does not influence these parameters [44]. Although the analysis of the influence of steatosis on IVIM components was hampered due to the low number of patients without steatosis (5 patients), our results point towards the degree of steatosis to be a possible confounder factor for D and D*.

The individual influence of steatohepatitis and fibrosis could not be identified in this study due to the overlap between these groups in 16 patients. However, these conditions occur simultaneously in NAFLD spectrum. The first stage is isolated steatosis, which can be followed by steatohepatitis. Some patients with NASH will also develop fibrosis, and a minor group will progress to cirrhosis. Future studies with a larger number of patients can be performed to determine the individual influence of steatohepatitis and fibrosis on IVIM components.

This study had some limitations. The number of patients was not very large and the individual influence of steatohepatitis and fibrosis on the different components of IVIM DWI could not be determined. However, in NAFLD, these conditions overlap. The group with normal liver (no steatosis) had only 5 patients. However, diabetic patients have a high prevalence of NAFLD and this finding is inherent of the chosen population. In addition, this study involved liver biopsy, an invasive method, associated with morbimortality risks. Considering previous studies that used histopathological analyses, this study has a large sample. Although care was taken to perform the best MRI acquisition possible, artifacts and low signal-to-noise images required that 14 patients had to be withdrawal from the study because of low quality images, a limitation inherent to the current magnets available for clinical research.

In conclusion, our results showed significantly lower D and D* in patients with NASH. IVIM diffusion was also useful for the detection of fibrosis. These results point to its future use as a non-invasive tool for the diagnosis of the severity of NAFLD.
